# Electromagnetic Dosimetry for Isolated Mitochondria Exposed to Near‐Infrared Continuous‐Wave Illumination in Photobiomodulation Experiments

**DOI:** 10.1002/bem.22342

**Published:** 2021-05-18

**Authors:** Andrea Amaroli, Stefano Benedicenti, Bruno Bianco, Alessandro Bosco, Mario Rene Clemente Vargas, Reem Hanna, Praveen Kalarickel Ramakrishnan, Mirco Raffetto, Silvia Ravera

**Affiliations:** ^1^ Department of Surgical and Diagnostic Sciences University of Genoa Genoa Italy; ^2^ Department of Orthopaedic Dentistry I.M. Sechenov First Moscow State Medical University Moscow Russian Federation; ^3^ Department of Electrical, Electronic, Telecommunications Engineering and Naval Architecture University of Genoa Genoa Italy; ^4^ Bosco Ottica S.r.l. Bergamo Italy; ^5^ Department of Oral Surgery King's College Hospital NHS Foundation Trust London UK; ^6^ Department of Experimental Medicine University of Genoa Genoa Italy

**Keywords:** low‐level light therapy, near‐infrared band, electromagnetic field inside mitochondria, average energy and dissipated power densities, numerical simulations, electromagnetic dosimetry

## Abstract

This paper presents results on the electromagnetic field computed inside isolated mitochondria when they are exposed to near‐infrared illuminations with reference to photobiomodulation experiments. The accurate calculation of the electromagnetic dose is considered to be important for a better understanding of the mechanism of interaction of light with these organelles and to improve the reliability and repeatability of the experiments. To get such results, we introduce several models. Even though they refer to a well‐defined experimental setup, different models are necessary to take into account the possible different dispositions of the mitochondria, and of the differences in their dimensions and in their constitutive parameters. Different wavelengths and polarizations are considered as well. The effects of all parameters on the electromagnetic field inside mitochondria are discussed. © 2021 Bioelectromagnetics Society.

## INTRODUCTION

Photobiomodulation (PBM), also known as low‐level light therapy (LLLT), was initially developed as a therapeutic procedure, and nowadays its importance is well‐established as beneficial in the treatment of human patients, such as in terms of inflammation reduction or to promote healing of wounds [Posten et al., 2005; Chung et al., 2012]. A large set of references reporting PBM effects is now available [Zein et al., 2018; and the references therein]. The therapy consists of irradiating the biological material of interest with narrow‐band electromagnetic fields in the visible or near‐infrared (NIR) portions of the spectrum [Chung et al., 2012].

Experimental evidence [Karu, 2008; De Freitas and Hamblin, 2016; Denton et al., 2019] shows that very important reactions take place inside mitochondria due to PBM [Scheffler, 2008]. The same was proven by experiments involving mitochondria in vitro [Passarella and Karu, 2014].

In Hadis et al. [2016], the authors describe the problems related to the miscalculation of the electromagnetic dose and the resulting issues with the reliability and repeatability of some of the PBM experiments. We try to undertake a quantitative study of the electromagnetic fields involved in properly defined PBM experiments that can help avoid such problems. In spite of the large set of experimental investigations and numerous explanations, the mechanism of the light interaction with biological targets is not fully understood [Passarella and Karu, 2014; Amaroli et al., 2016]. In order to get closer to this result, it has been observed [Hadis et al., 2016] that it is necessary to have a better knowledge of the electromagnetic field inducing the biological reactions.

The target of our work is to increase such knowledge. Unfortunately, electromagnetic simulations of realistic problems, even the simplest ones related to in vitro experiments, require rather complex calculations. This is due to several reasons. Some of these are related to the incubation chambers where mitochondria are placed to carry out the experiments of interest. As a matter of fact, their dimensions are huge with respect to the wavelength, and their geometry may present joined cylindrical and conical sections, each extending for several millimeters [Passarella and Karu, 2014; Amaroli et al., 2016]. The laser source can introduce additional complexities to models of realistic experiments. Since it usually generates a Gaussian beam, whose intensity changes with the distance from the source itself and from the center of the beam in the transverse direction [Van Bladel, 2007], it can determine different conditions of illumination of the biological organelles. Finally, in the electromagnetic simulations one has to cope with the fact that the dispositions of the many mitochondria involved in experiments are rather random, that the same is true for their dimensions, and that, moreover, their internal features may change, in a partially unknown way, from one mitochondrion to the other [Scheffler, 2008].

In this paper, we overcome some of these difficulties by considering models, as usual in electromagnetic dosimetry [Durney, 1980; Chou et al., 1996; International Commission on Non‐Ionizing Radiation Protection (ICNIRP), 2020]. All our numerical models refer to a well‐defined experimental arrangement, involving an ad hoc incubation chamber and a laser with a flat‐top handpiece [Amaroli et al., 2016]. This helps to simplify the geometry of the chamber and to ensure uniform illumination of the chamber itself. Moreover, by adopting the approach proposed by several studies that dealt with the determination of the dielectric features of mitochondria in the NIR band, we consider them as made up of a homogeneous effective medium [Beauvoit et al., 1994; Ullah et al., 2010]. Thus, all mitochondria involved in our models are simulated by homogeneous ellipsoids having the same dimensions and the same value of a complex refractive index.

In spite of the indicated simplifications, our analysis is still complex. To face the random disposition of mitochondria and the variability of their geometrical and constitutive parameters, we introduce several three‐dimensional models, taking account of the possible different alignments (horizontal or vertical) and configurations (isolated, layered, or clustered), which mitochondria can assume in practice. Moreover, for each numerical model, several values of their dimensions and constitutive parameters are considered. Finally, different wavelengths and polarizations are used.

Several methods have been adopted in the literature to compute the electromagnetic fields in the presence of biological cells or organelles [Dunn and Richards‐Kortum, 1996; Saho et al., 2001; Karlsson et al., 2005; Yu et al., 2013]. To the best of our knowledge, there are no results in the open literature related to the computation of the electromagnetic fields inside the biological organelles exposed to the wavelengths pertinent to PBM. In this work, the finite element method [Jin, 1993] is used for solving the three‐dimensional time‐harmonic Maxwell's equations [Harrington, 1961] along with the appropriate boundary conditions to compute the fields of interest.

The effects of possible changes are analyzed and discussed, for the first time to the best of the authors' knowledge, in terms of the quantities of interest for the PBM of mitochondria in vitro. In particular, the behavior of the field inside mitochondria, its average energy, and the dissipated power densities are studied in detail.

## MATERIALS AND METHODS

As mentioned above, the electromagnetic simulations for determining the fields inside the biological organelles are extremely challenging. The difficulties arise due to various reasons, such as the large size of the domain in terms of the wavelength, the non‐uniform illumination from laser beams, and the variability of the geometric and optical parameters of the organelles. We try to overcome these challenges by proposing a careful experimental setup that allows us to develop a manageable electromagnetic model. This is done by considering mitochondria placed at the bottom of a cylindrical incubation chamber filled with saline solution and a continuous‐wave laser source with a flat‐top handpiece. The setup simplifies the geometry of the problem and ensures uniform illumination of the organelles as described in the first subsection below. Further, the three‐dimensional modeling of the electromagnetic problem is achieved by exploiting the fact that mitochondria placed in the saline solution are weak scatterers, and hence the electromagnetic fields at a relatively small distance from them are not significantly affected due to their presence. Thus, we are able to consider various three‐dimensional models in a smaller domain that is computationally manageable as described in the second subsection below. The final three‐dimensional model is then solved using a commercial electromagnetic solver as described in the third subsection.

### The Proposed Experimental Setup

**Fig. 1:**
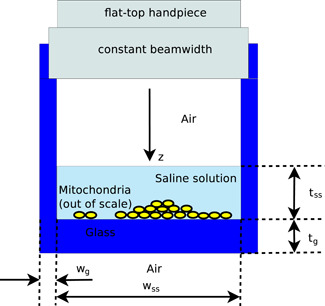
The simple experimental setup to which all our models refer. A laser with a flat‐top handpiece is used to generate a beam, uniform in width, which illuminates a set of mitochondria at the bottom of an incubation chamber made of glass.

The quantification of the electromagnetic field in mitochondria in vitro exposed to optical or NIR illumination will be carried out by using models, which is usual in electromagnetic dosimetry [Durney, 1980; Chou et al., 1996; ICNIRP, 2020]. Such models, which will be introduced in the next subsection, are all related to the experimental setup shown in Figure 1. We plan to undertake the experiment with this setup following the present numerical study.

The proposed setup closely follows the experiments described in Amaroli et al. [2016] but with properly defined geometry and optical properties of the materials that will allow us to derive a manageable electromagnetic model. In the simple setup reported in Fig. 1, a flat‐top handpiece [Amaroli et al., 2016] is hosted in the incubation chamber made of glass. It generates a beam, uniform in width, which illuminates with a uniform intensity the media below it (see, for example, Fig. 1 of Amaroli et al., 2016). In order to operate under the above‐said conditions, the incubation chamber has to be cylindrical, with a circular cross‐section of area $≅1\;{\mathrm{cm}}^{2}$ [Amaroli et al., 2016], corresponding to a diameter $w_{\mathrm{ss}}≅1.13\;\mathrm{cm}$. We have to avoid much larger diameters because, on the one hand, the flat‐top handpiece is designed to guarantee a spot size of 1 cm^2^ and, on the other hand, we would like to uniformly illuminate the mitochondria, which generally spread over all the extension of the floor. At the same time, we have to refrain from using a smaller cross‐section to fully exploit the capabilities of a flat‐top handpiece and to avoid strong scattering effects from the vertical walls of the incubation chamber.

As it is shown in Fig. 1, in the incubation chamber we can find air, a saline solution, and at the bottom of the chamber, a set of mitochondria. The medium below the glass of the chamber is air and it has to be ensured that there are no obstacles that may cause the outgoing wave to be reflected back into the chamber.

The considered saline solution contains 0.1 M Tris‐HCl (pH 7.4), 0.1 M KCl, 5 mM MgCl_2_, 0.2 mM P1,P5‐Di(adenosine‐5') pentaphosphate, 0.6 mM ouabain, and 5 mM KH_2_PO_4_. The molarity of this solution is small and is very close to the smallest value of the concentrations considered in Wang et al. [2017]. This reference shows that, for the concentration and wavelengths of our interest, the complex refractive index of several binary and mixed‐salt solutions presents negligible difference with respect to that of deionized water or standard saline solutions. For this reason, we will assume that the refractive index of our solution is the same as that of simple water [see also Morel, 1974; Pegau et al., 1997).

In the usual process, mitochondria are isolated from the bovine liver by a standard differential centrifugation technique [Ravera et al., 2011] and are suspended in the solution. After a while, they spread out at the bottom of the incubation chamber because mitochondria have a mass density in the range [1.09, 1.35] g/cm^3^ [Kim et al., 2017], which is larger than that of the saline solution, being equal to 1.02 g/cm^3^.

The layer of mitochondria at the bottom of the chamber should not be too thick so as to ensure that the resulting electromagnetic model is not too complex. The quantity of organelles involved in experiments is usually expressed in terms of the mass of the mitochondria proteins [Amaroli et al., 2016]. Considering, for example, 50 μg of such proteins [Amaroli et al., 2016] and taking account of an average protein concentration of 0.5 g/ml [Thar and Kühl, 2004], we obtain an overall volume of mitochondria of $\frac{5\times 10^{-5}}{0.5}=10^{-4}$ ml, corresponding to an average thickness at the bottom of the incubation chamber of 1 μm. Even if the final disposition of the organelles on the 1 cm^2^ floor is not regular, we can conclude that it is unlikely to have to deal with clusters of mitochondria having a height larger than very few micrometers.

### Electromagnetic Modeling of the System for the Computation of the Fields Inside Mitochondria

As we have already pointed out, in the simple experimental setup to which we refer, the illuminating field is generated by a laser and comes out of a flat‐top handpiece that is able to guarantee a very good uniformity of the illumination in a way largely independent of the distance of the handpiece from the target. For these reasons, we can consider, with a very good approximation, that the content of the incubation chamber is illuminated by a monochromatic uniform plane wave propagating along the z direction (see Fig. 1).

Such a monochromatic uniform plane wave, whose time‐harmonic dependence factor $e^{j\omega t}$is assumed and suppressed throughout (ω is the angular frequency and t is time) [Harrington, 1961], propagates inside the incubation chamber. When no mitochondria are present, it interacts with a multilayer structure of plane layers made up, respectively, of air, saline solution, glass, and again air, provided that we avoid considering the regions very close to the vertical walls of the chamber. This problem can be easily solved by an analytical procedure for multilayer structures [Orfanidis, 2016].

**Fig. 2:**
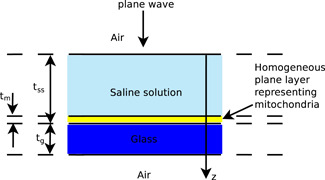
A rough one‐dimensional model where the mitochondria are replaced by an effective homogeneous plane layer.

The same tool can be used to solve a one‐dimensional model of the problem of interest in which the mitochondria are replaced by an effective homogeneous plane layer (see Fig. 2). Such a model is a rough approximation of the experimental setup we consider (see Fig. 1) and can be exploited just to give preliminary indications on the magnitudes of the quantities of interest. The thicknesses of the saline solution, $t_{\mathrm{ss}}$, and of the glass, $t_{g}$, together with their complex refractive indices, $n_{\mathrm{ss}}$, $n_{g}$, are known quantities for all wavelengths of interest. On the contrary, the thickness and the refractive index, $t_{m}$ and $n_{m}$, of the plane layer representing the mitochondria have to be considered as variable quantities, to be able to analyze different situations of potential interest.

For a generic complex refractive index n we will use the convention $n=n^{\prime }-\mathrm{jn}\prime \prime$, $n^{\prime }>0$, $n^{\prime \prime }\geq 0$. Very often we will define $n^{\prime \prime }$ by using the corresponding absorption coefficient $\mu_{a}$ (m^−1^), since $\mu_{a}=\frac{4\pi n^{\prime \prime }}{\lambda_{0}}$ [Jacques, 2013], $\lambda_{0}$ being the wavelength in vacuum.

The best models for the evaluation of the electromagnetic field within mitochondria are of course three‐dimensional. Unfortunately, with present‐day commercial simulators and computers, it is not possible to consider models with all details of the three‐dimensional scattering problem of interest (see again Fig. 1) because the corresponding domains of numerical investigations would be, in any case, huge with respect to $\lambda_{0}^{3}$. As a matter of fact, the usual values of $t_{g}$ and $t_{\mathrm{ss}}$ are of the order of a few millimeters. The flat‐top handpiece can be close to the air‐saline solution interface but, in any case, in order to take account of all horizontal interfaces, one has to consider a domain height of several millimeters. Moreover, the cross‐section of the incubation chamber is about 1 cm^2^. Thus, even while neglecting the vertical walls of the chamber one would have to deal with a domain of investigation of about 1 cm^3^. Considering that we are interested in $\lambda_{0}$ values of 808, 980, or 1064 nm, one easily understands that the domain of investigation could be as large as one thousand billion cubic wavelengths. Since in any discretization procedure a cubic wavelength requires approximately one thousand degrees of freedom, we conclude that any realistic three‐dimensional simulation should be able to deal with about 10^15^ unknowns, which is by far too large a number for present‐day computers and simulators.

Fortunately, one can avoid such a brute force approach by observing that, independently of our knowledge of the detailed internal structure of mitochondria, they are in any case weak scatterers. This is because they have dimensions of the same order of magnitude of the vacuum wavelength of interest for PBM [Scheffler, 2008] and, moreover, in terms of electromagnetic scattering they behave as homogeneous [Beauvoit et al., 1994; Ullah et al., 2010] ellipsoids having an estimated effective refractive index $n_{m}$ [Beauvoit et al., 1994; Thar and Kühl, 2004; Ullah et al., 2010], which is really close to the refractive indices of the saline solution and glass. For the saline solution [Palmer and Williams, 1974; Wang et al., 2017] and glass [Lentes et al., 1998], we can consider

- at $\lambda_{0}=808\;\mathrm{nm}$: $n_{\mathrm{ss}}^{\prime }=1.331$, $\mu_{a,\mathrm{ss}}=1.95$ m^−1^ ($n_{\mathrm{ss}}^{\prime \prime }=1.25\times 10^{-7}$), $n_{g}^{\prime }=1.511$, $\mu_{a,g}=0.170$ m^−1^ ($n_{g}^{\prime \prime }=1.09\times 10^{-8}$),
- at $\lambda_{0}=980$ nm: $n_{\mathrm{ss}}^{\prime }=1.328$, $\mu_{a,\mathrm{ss}}=50.2$ m^−1^ ($n_{\mathrm{ss}}^{\prime \prime }=3.91\times 10^{-6}$), $n_{g}^{\prime }=1.508$, $\mu_{a,g}=0.122$ m^−1^ ($n_{g}^{\prime \prime }=9.50\times 10^{-9}$),
- at $\lambda_{0}=1064$ nm: $n_{\mathrm{ss}}^{\prime }=1.328$, $\mu_{a,\mathrm{ss}}=16.2$ m^−1^ ($n_{\mathrm{ss}}^{\prime \prime }=1.37\times 10^{-6}$), $n_{g}^{\prime }=1.507$, $\mu_{a,g}=0.099$ m^−1^ ($n_{g}^{\prime \prime }=8.37\times 10^{-9}$),

while for mitochondria $n_{m}^{\prime }$ and $\mu_{a,m}$ are expected to be, respectively, in the ranges [1.35, 1.45] and [20, 150] m^−1^ (*n*″_*m*_ ∈ [1.28 10^−6^, 9.61 10^−6^])  at $\lambda_{0}=800\;\mathrm{nm}$ [Beauvoit et al., 1994; Thar and Kühl, 2004; Ullah et al., 2010] and the same ranges for $n_{m}^{\prime }$ and $\mu_{a,m}$ are retained at 980 and 1064 nm.

Since the field in a mitochondrion is not affected by the presence of distant mitochondria, in our models, we can consider the presence of small numbers of scattering mitochondria. The few weakly interacting mitochondria considered in our three‐dimensional models generate the so‐called scattered field [Monk, 2003], which becomes negligible with respect to the so‐called incident field (i.e., the field when no mitochondria are present [Monk, 2003]) a small distance away from the scatterers. For this reason, we will approximate the scattering problems of interest by enforcing inhomogeneous impedance boundary conditions [Monk, 2003; Fernandes and Raffetto, 2005; Fernandes and Raffetto, 2009; Kalarickel Ramakrishnan and Raffetto, 2020] on a surface enclosing the few mitochondria we will consider. The negligible amplitude of the scattered field on such a surface allows us to calculate the inhomogeneous term of the impedance boundary condition by using the incident field, which is provided by the analytical procedure described above [Monk, 2003].

**Fig. 3:**
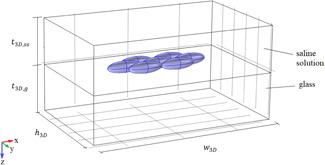
A three‐dimensional model with seven mitochondria arranged in a single layer in the horizontal position.

**Fig. 4:**
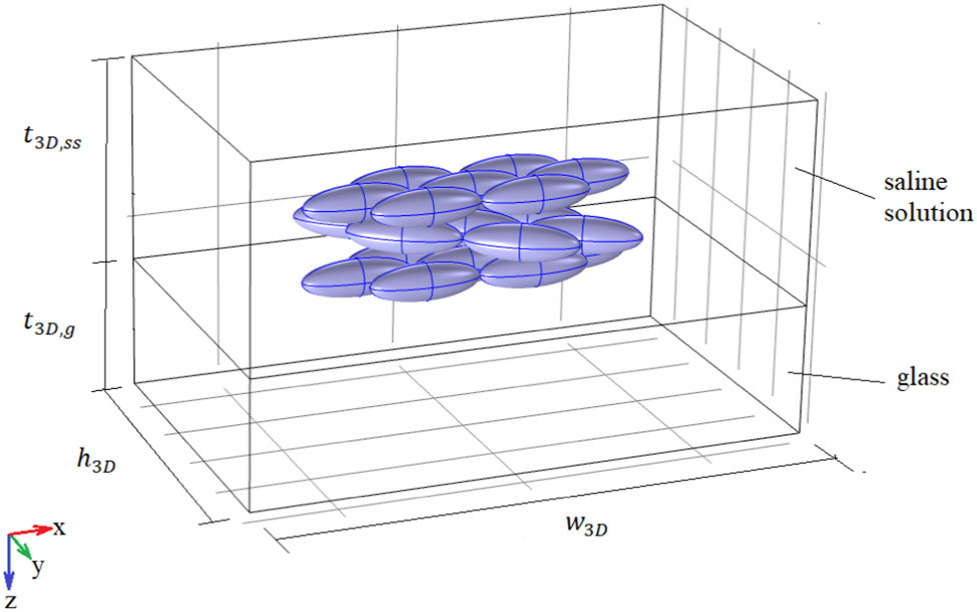
A three‐dimensional model with 21 mitochondria arranged in three layers in the horizontal position.

One example of the models we consider is reported in Fig. 3, where a mitochondrion is surrounded by six other similar organelles. In a single layer horizontal disposition of mitochondria, it is not necessary to consider other organelles, due to the weakness of their scattering effects. With the same thickness, we will also consider for comparison an isolated mitochondrion and a periodic arrangement of such organelles (just in this case the impedance boundary conditions on the lateral walls will be replaced by periodic boundary conditions [Itoh, 1989]). The effects of a larger height of the region occupied by mitochondria will be quantitatively analyzed by considering either a group of seven mitochondria, all placed in vertical positions, or twenty‐one mitochondria, organized as shown in Figure 4. In particular, in such a model the mitochondria of the middle layer can have the same orientation as those of the other layers or be rotated by a 90° angle (no other angle values are considered in order to avoid dealing with too many models). Taking account of the very limited average height of the region occupied by mitochondria at the bottom of the incubation chamber, we do not consider other three‐dimensional models.

### Details of the Electromagnetic Simulations

The models defined in the previous section are exploited to evaluate the electromagnetic field inside mitochondria. Their length, $l_{m}$, is set to 3 μm while their diameter, $d_{m}$, can be equal to 0.5, 0.75, or 1 μm [Scheffler, 2008]. The thickness of the saline solution, $t_{\mathrm{ss}}$, is fixed at 5 mm and that of the glass, $t_{g}$, at 2 mm. The axes directions are shown in Figure 3 and the origin is placed on the air–saline solution interface.

In all simulations, we have always considered an impinging monochromatic plane wave having a power density of 1 W cm^−2^ (corresponding to an amplitude of the electric field, |E|, of 2744.9 V m^−1^) and a wavelength of 980 nm unless otherwise specified.

All the results related to three‐dimensional models that we present in this section have been calculated by using COMSOL Multiphysics (COMSOL, Burlington, MA), a commercial simulator based on the finite element method [Jin, 1993]. The simulations have been performed on an HP Z240 workstation, equipped with an Intel core i7‐7700 quad‐core processor and 64 GB of RAM memory.

In terms of quality of the approximation, we have obtained good results when the minimum distance of the boundary from the scatterers is larger than or equal to ${1.5\lambda }_{0}$. The results we present, however, have been obtained by using a distance of at least ${2.5\lambda }_{0}$, to have a good margin of safety. These considerations do not apply to the model considering a periodic arrangement of mitochondria, since in that case, the distance can be much smaller without affecting the quality of the approximation.

## RESULTS

### Effects of the Polarization of the Incident Field

**Fig. 5:**
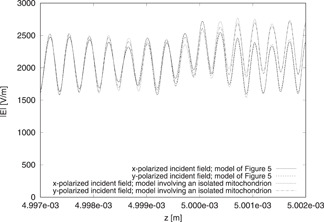
Behavior of |E| along the *z* axis. The results are computed by using either the model involving an isolated mitochondrion or that of Figure 3 for different linear polarizations.

In this subsection, the incident field is assumed to be linearly polarized along the *x* or *y* axis. In Figure 5, we show the magnitude of the electric field along the *z* axis in the presence of either an isolated mitochondrion or seven horizontal mitochondria (model of Fig. 3). The results are calculated for $n_{m}^{\prime }=1.4$ and *μ_a,m_* = 85 m^−1^, which are the middle values of the ranges indicated in the previous section, and *d_m_* =1 μm.

The mitochondria in these models are in the range (4.999, 5) mm. The *z* axis passes through the center of gravity of the central mitochondrion of the models considered.

The solutions present some differences, which are not large with respect to the average values of the magnitudes of the fields, but if one focuses, in particular, on |E| in the central mitochondrion, one can see that the effects due to the different polarizations of the incident field are negligible. The very small differences in terms of fields in the mitochondria result in negligible differences in terms of average (in space and time) energy density of the electromagnetic field [Orfanidis, 2016]

(1) $$E_{d}=\frac{1}{V_{m}}\int_{V}\frac{1}{4}(\epsilon_{0}\epsilon_{r,m}^{'}{|E|}^{2}+\mu_{0}{|H|}^{2})$$

and of average (in space and time) dissipated power density in the central mitochondrion (occupying the region $V_{m}$ in the formulas) [Orfanidis, 2016]

(2) $$P_{d}=\frac{1}{V_{m}}\int_{V}\frac{1}{2}\omega \epsilon_{0}\epsilon_{r,m}^{\prime \prime }{|E|}^{2}$$

where $\epsilon_{r,m}^{'}$ and $\epsilon_{r,m}^{\prime \prime }$ are, respectively, the real part and the magnitude of the imaginary part of the relative permittivity of the mitochondrion, $\epsilon_{r,m}$, being $\epsilon_{r,m}=\epsilon_{r,m}^{'}-j\epsilon_{r,m}^{\prime \prime }=n_{m}^{2}$. The effects due to dispersion for the media involved were verified to be negligible at the wavelengths of interest and hence the equation corresponding to non‐dispersive media is used for $E_{d}$.

To generalize this conclusion, we computed $E_{d}$ and $P_{d}$ by using different polarizations and models. For all models, the results obtained by considering either the *x* or *y* polarization of the incident field are almost the same, with a maximum difference for $E_{d}$ of less than 1.3% and for $P_{d}$ of less than 2.7%. In the subsection after the next, it will be shown that the dimensions and constitutive parameters of the mitochondria are not able to affect this result in a significant way. Therefore, in general, we can conclude that for studies related to the PBM of mitochondria in vitro, the polarization has negligible effects. Taking account of this conclusion, all the following results are calculated by using the y polarization of the incident field.

### Comparison of One‐ and Three‐Dimensional Results

The previous considerations do not exclude the possibility of getting fairly good approximations from one‐dimensional models. For this reason, we now compare the outcomes of the one‐dimensional model of Figure 2 with those of the more detailed three‐dimensional models. In this way, we hope to be able to draw some conclusions about the quality of the results provided by the simplest model.

**Fig. 6:**
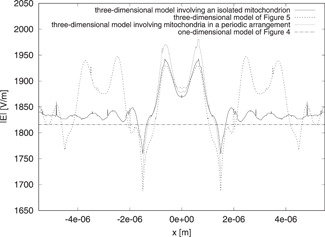
Behavior of |E| along a line parallel to the *x* axis and passing through the center of gravity of the mitochondria. The results are computed by using different one‐dimensional and three‐dimensional models.

To illustrate the principle, we fix the value of the parameters by considering again mitochondria with *d_m_* = 1 μm, $n_{m}^{\prime }=1.4$, and *μ_a,m_* = 85 m^−1^. Correspondingly, we set $t_{m}=1$ μm for the one‐dimensional model. In Figure 6, the magnitudes of the total electric fields are plotted along a line parallel to the *x* axis and passing through the center of gravity of the central mitochondrion. The one‐dimensional approximation is compared against the results obtained from the three‐dimensional models involving either one mitochondrion (isolated or in a periodic arrangement) or seven mitochondria in a horizontal disposition. Although the one‐dimensional model cannot provide the details about the spatial variations of the fields, the results obtained using it can nevertheless be a good first approximation to those obtained from the more accurate models. In particular, the maximum difference between the fields of the three‐dimensional models with respect to that of the one‐dimensional one is less than 6.3% of the incident field magnitude.

**Fig. 7:**
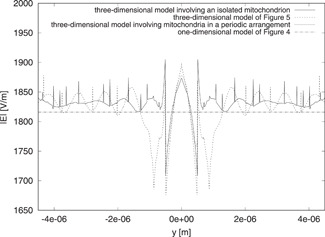
Behavior of |E| along a line parallel to the *y* axis and passing through the center of gravity of the mitochondria. The results are computed by using different one‐dimensional and three‐dimensional models.

Similarly, in Figure 7, the fields for the different models are compared along a line parallel to the *y* axis and passing through the central mitochondrion. The one‐dimensional result is close to all three three‐dimensional solutions, giving a maximum difference of less than 5.2% of the incident field. We do not plot the fields along the *z* axis because the results are very similar to those shown in Fig. 5. The fields, in this case too, are comparable, although there is a maximum difference of about 12.6% in the glass. However, in the saline solution and inside mitochondria, the difference is not larger than 3.7% of the incident field.

As before, these considerations can be extended to the averages of energy and dissipated power densities. The $E_{d}$ value given by the one‐dimensional model differs from that obtained by the three‐dimensional models involving either one isolated mitochondrion or seven mitochondria in a horizontal disposition, respectively, by 4.94% and 2.63%. Likewise, the $P_{d}$ value differs by 4.08% and 1.78% in the two cases, respectively.

A similar comparison is possible between the one‐dimensional model with $t_{m}=3$ μm and the three‐dimensional models having the same height of the region occupied by mitochondria (namely that shown in Fig. 4 or the one involving seven mitochondria in a vertical disposition). For example, for the same $n_{m}^{\prime }$ and $\mu_{a,m}$ considered above, the difference between the $E_{d}$ values obtained from the one‐dimensional model and the three‐dimensional one of Figure 4 is just 1.61% and the corresponding $P_{d}$ difference is 1.72%. The same differences, but evaluated in the regions occupied by the central mitochondrion of the top and bottom layers, are respectively 1.05% and 2.68% for the average energy density and 0.49% and 1.73% for the average dissipated power density. As for the comparison between the values of the one‐dimensional model and the other three‐dimensional models considered here, the averages of energy density and power density differ, respectively, by 7.77% and 7.78%.

In summary, one can observe that the one‐dimensional model can be used to obtain fast results that are good estimates for the quantities of interest. Moreover, the results of the next subsection show that the same conclusion holds true for all values of the parameters. However, for a more accurate evaluation of the fields, we need to make use of the three‐dimensional models.

### Effects of Geometrical and Constitutive Parameters

**Fig. 8:**
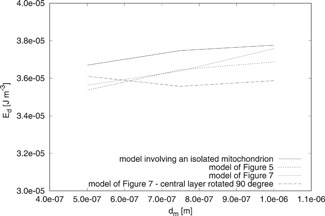
Behavior of $E_{d}$ for $d_{m}\in \{0.5,0.75,1\}$ μm. The constitutive parameters of the mitochondria are fixed ($n_{m}^{\prime }=1.4$, $\mu_{a,m}=85\;m^{-1}$), but different three‐dimensional models are considered to compute $E_{d}$.

**Fig. 9:**
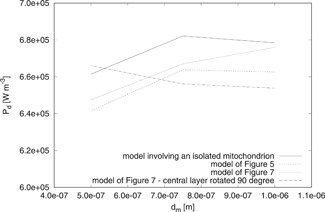
Behavior of $P_{d}$ for $d_{m}\in \{0.5,0.75,1\}$ μm. The constitutive parameters of the mitochondria are fixed ($n_{m}^{\prime }=1.4$, $\mu_{a,m}=85\;m^{-1}$), but different three‐dimensional models are considered to compute $P_{d}$.

A couple of the above deductions referred to the effects of the parameters $d_{m},n_{m}^{\prime }$, and $\mu_{a,}$ in our results. In Fig. 8, the $E_{d}$ values calculated by using the three‐dimensional models of Figs. 3 and 4 together with that involving an isolated mitochondrion are shown. They are calculated by considering $n_{m}^{\prime }$ and $\mu_{a,m}$ as fixed quantities ($n_{m}^{\prime }=1.4$ and *μ_a,m_* = 85 m^−1^) while $d_{m}$ is free to change in the set {0.5,0.75,1} μm [Scheffler, 2008]. Figure 9 reports the analogous behavior of $P_{d}$.

One can observe that $d_{m}$ does not affect the values of $E_{d}$ and $P_{d}$ in a significant way. The largest of these effects is smaller than 5%. Even though we do not show the results, we have verified that the conclusion holds true for all of the considered $n_{m}^{\prime }$ and $\mu_{a,m}$ values.

We now analyze the effects of $n_{m}^{\prime }$ and $\mu_{a,m}$ on $E_{d}$ and $P_{d}$ by fixing $d_{m}=1$ μm. The results are summarized in Tables 1 and 2.

**Table 1:** Values of $E_{d}$ (J m^−3^) computed by using different values of $n_{m}^{\prime }$ and $\mu_{a,m}$ and different three‐dimensional models

	Model with an isolated mitochondrion	Model Figure 5	Model Figure 7
$n_{m}^{\prime }=1.35$	$3.55\times 10^{-5}$	$3.52\times 10^{-5}$	$3.54\times 10^{-5}$
$n_{m}^{\prime }=1.40$	$3.78\times 10^{-5}$	$3.69\times 10^{-5}$	$3.76\times 10^{-5}$
$n_{m}^{\prime }=1.45$	$4.01\times 10^{-5}$	$3.87\times 10^{-5}$	$4.05\times 10^{-5}$

The results are independent of *μ_a,m_*.

**Table 2:** Values of $P_{d}$ (W m^−3^) computed by using different values of $n_{m}^{\prime }$ and $\mu_{a,m}$ and different three‐dimensional models

	Model with an isolated mitochondrion	Model Figure 5	Model Figure 7
$n_{m}^{\prime }=1.35$, $\mu_{a,m}=20\;m^{-1}$	$1.56\times 10^{5}$	$1.55\times 10^{5}$	$1.56\times 10^{5}$
$n_{m}^{\prime }=1.35$, $\mu_{a,m}=85\;m^{-1}$	$6.65\times 10^{5}$	$6.59\times 10^{5}$	$6.62\times 10^{5}$
$n_{m}^{\prime }=1.35$, $\mu_{a,m}=150\;m^{-1}$	$1.17\times 10^{6}$	$1.16\times 10^{6}$	$1.17\times 10^{6}$
$n_{m}^{\prime }=1.40$, $\mu_{a,m}=20\;m^{-1}$	$1.60\times 10^{5}$	$1.56\times 10^{5}$	$1.59\times 10^{5}$
$n_{m}^{\prime }=1.40$, $\mu_{a,m}=85\;m^{-1}$	$6.78\times 10^{5}$	$6.63\times 10^{5}$	$6.76\times 10^{5}$
$n_{m}^{\prime }=1.40$, $\mu_{a,m}=150\;m^{-1}$	$1.20\times 10^{6}$	$1.17\times 10^{6}$	$1.19\times 10^{6}$
$n_{m}^{\prime }=1.45$, $\mu_{a,m}=20\;m^{-1}$	$1.63\times 10^{5}$	$1.57\times 10^{5}$	$1.66\times 10^{5}$
$n_{m}^{\prime }=1.45$, $\mu_{a,m}=85\;m^{-1}$	$6.94\times 10^{5}$	$6.69\times 10^{5}$	$7.05\times 10^{5}$
$n_{m}^{\prime }=1.45$, $\mu_{a,m}=150\;m^{-1}$	$1.22\times 10^{6}$	$1.18\times 10^{6}$	$1.24\times 10^{6}$

As expected from our former considerations, the differences of the values computed by using different models are negligible. Moreover, as a consequence of Equations (1) and (2) and the relation between $n_{m}$ and $\epsilon_{r,m}$, $E_{d}$ results to be totally independent of $\mu_{a,m}$ and increases with $n_{m}^{\prime }$ while $P_{d}$ is largely independent of $n_{m}^{\prime }$ and is directly proportional to $\mu_{a,m}$. This can happen because the indicated changes of the constitutive parameters are not able to modify the average value of |E| in the central mitochondrion in a significant way.

Based on these results, we can say that the variabilities of the indicated parameters in the considered ranges have small effects on the fields stimulating the mitochondria during the PBM experiments, and the dissipated power density is directly proportional to $\mu_{a,m}$.

### Analysis of the Scattered Field

In defining most of our three‐dimensional models, we have assumed that the mitochondria are weak scatterers and that it is not necessary to consider the organelles that are far away, in the planes orthogonal to the direction of propagation of the incident field, from the ones under investigation. In this subsection, we provide some justifications for this assumption.

Let $E_{i}$ be the electric field of the incident wave, which is obtained when no mitochondrion is present (as already pointed out in the Section “Materials and Methods”). We define, as usual, the scattered field, $E_{s}$, as the difference between the electric field in the presence of mitochondria and $E_{i}$ [Monk, 2003].

**Fig. 10:**
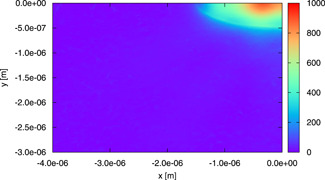
A color image of $\left|E_{s}\right|$ on the plane z=4999.5 μm passing through the center of gravity of the mitochondrion. The field is computed for the model involving an isolated mitochondrion by using $d_{m}=1$ μm, $n_{m}^{'}=1.45$, and *μ_a,m_* = 150 m^−1^.

**Fig. 11:**
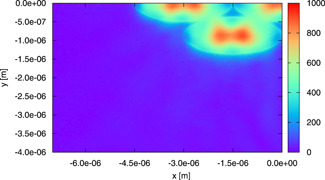
A color image of $\left|E_{s}\right|$ on the plane z=4999.5 μm passing through the center of gravity of the mitochondria. The field is computed for the model of Figure 3 by using $d_{m}=1$ μm, $n_{m}^{'}=1.45$, and *μ*_*a,m*_ = 150 m^−1^.

In Figs. 10 and 11, we show the color images of $\left|E_{s}\right|$ on the plane z=4999.5 μm passing through the center of gravity of the central mitochondria (just a quarter of the plane is shown for all figures of this type). The fields are computed, respectively, by using the model involving an isolated mitochondrion and that of Fig. 3 and all the organelles are characterized by $d_{m}=1$ μm, $n_{m}^{\prime }=1.45$, and *μ_a,m_* = 150 m^−1^, to get the largest scattering effect.

If one focuses on the scattered field, which is present in the central mitochondrion, one can notice, by comparison, that the neighboring mitochondria are not strongly influencing it. Hence, we can conclude that the lateral interaction is weak and that our models, which consider a small number of mitochondria in the same plane, are correct. Moreover, in the indicated plane, $\left|E_{s}\right|$ is not negligible just inside mitochondria. From this consideration, we can understand why it is not necessary to place the lateral boundaries far away from the organelles.

**Fig. 12:**
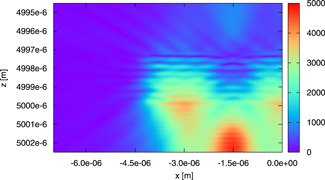
A color image of $\left|E_{s}\right|$ on the (x,z) plane (passing through the center of gravity of the central mitochondrion of each layer). The field is computed for the model in Figure 4 by using $d_{m}=1$ μm, $n_{m}^{'}=1.45$, and *μ_a,m_* = 150 m^−1^.

**Fig. 13:**
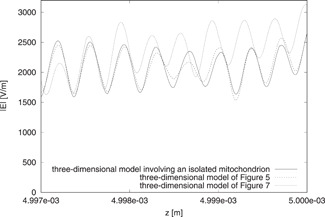
Behavior of |E| along the *z* axis. The results are computed by using the model involving an isolated mitochondrion and those of Figures 3 and 4.

In Fig. 12, we show $\left|E_{s}\right|$ on the (x,z) plane for the model of Fig. 4. The same values of $d_{m}$, $n_{m}^{\prime }$, and $\mu_{a,m}$ as before are used. Since $E_{s}$ does not present a significant standing wave pattern in the z direction, we understand that the top and bottom walls of the boundary are able to absorb such a field. Moreover, Fig. 12 shows that the presence of three layers affects the field. This is confirmed by the behavior of |E| along the *z* axis, which is reported in Fig. 13. However, it can be observed that |E| in the central mitochondrion of the top layer of the model of Figure 4 is similar to the same quantity in the corresponding mitochondria of the other models considered in the figure. The results are not so similar when the central and bottom layers of the model of Figure 4 are considered, but in any case, the difference is not too large. In terms of average energy and dissipated power densities in the central mitochondrion of the different layers, the results are almost identical. In particular, the average energy density (J m^−3^) in the central mitochondrion is equal to $3.78\times 10^{-5}$ for the model involving an isolated mitochondrion, $3.69\times 10^{-5}$ for the model of Fig. 3, and $3.66\times 10^{-5}$, $3.76\times 10^{-5}$, and $3.80\times 10^{-5}$ for the top, center, and bottom layer of the model in Fig. 4. In terms of average dissipated power density (W m^−3^), the corresponding numbers are $6.78\times 10^{5}$, $6.63\times 10^{5}$, $6.68\times 10^{5}$, $6.76\times 10^{5}$, and $6.82\times 10^{5}$.

### Different $\lambda_{0}$ Values

All the above outcomes were computed by using $\lambda_{0}=980\;\mathrm{nm}$. However, all the conclusions of the previous subsections hold true for $\lambda_{0}$ equal to 808 and 1064 nm.

Just to provide a few results to support this conclusion, in Tables 3 and 4, we report the $P_{d}$ values, respectively, for $\lambda_{0}=808$ and 1064 nm. The results were computed by using different models and different geometrical or constitutive parameters. These data confirm, for example, the quality of the approximation provided by the one‐dimensional model, and that $P_{d}$ is largely independent of $n_{m}^{\prime }$ and is directly proportional to $\mu_{a,m}$.

**Table 3:** Values of $P_{d}$ (W m^−3^) computed by using $\lambda_{0}=808$ nm, different models, and different geometrical or constitutive parameters

	Model Figure 4 *t_m_* = 1 μm	Model Figure 4 *t_m_* = 3 μm	Model with an isolated mitochondrion	Model Figure 5	Model Figure 7
$n_{m}^{\prime }=1.35$, $\mu_{a,m}=20\;m^{-1}$	2.12 × 10^5^	$2.01\times 10^{5}$	$2.17\times 10^{5}$	$2.15\times 10^{5}$	$2.16\times 10^{5}$
$n_{m}^{\prime }=1.35$, $\mu_{a,m}=85\;m^{-1}$	9.00 × 10^5^	$8.55\times 10^{5}$	$9.22\times 10^{5}$	$9.16\times 10^{5}$	$9.18\times 10^{5}$
$n_{m}^{\prime }=1.35$, $\mu_{a,m}=150\;m^{-1}$	1.59 × 106	$1.51\times 10^{6}$	$1.63\times 10^{6}$	$1.62\times 10^{6}$	$1.62\times 10^{6}$
$n_{m}^{\prime }=1.40$, $\mu_{a,m}=20\;m^{-1}$	2.03 × 105	$1.94\times 10^{5}$	$2.20\times 10^{5}$	$2.16\times 10^{5}$	$2.25\times 10^{5}$
$n_{m}^{\prime }=1.40$, $\mu_{a,m}=85\;m^{-1}$	8.61 × 105	$8.22\times 10^{5}$	$9.36\times 10^{5}$	$9.17\times 10^{5}$	$9.56\times 10^{5}$
$n_{m}^{\prime }=1.40$, $\mu_{a,m}=150\;m^{-1}$	1.52 × 106	$1.45\times 10^{6}$	$1.65\times 10^{6}$	$1.62\times 10^{6}$	$1.69\times 10^{6}$
$n_{m}^{\prime }=1.45$, $\mu_{a,m}=20\;m^{-1}$	1.98 × 10^5^	$2.16\times 10^{5}$	$2.22\times 10^{5}$	$2.17\times 10^{5}$	$1.96\times 10^{5}$
$n_{m}^{\prime }=1.45$, $\mu_{a,m}=85\;m^{-1}$	8.41 × 10^5^	$9.20\times 10^{5}$	$9.54\times 10^{5}$	$9.23\times 10^{5}$	$1.00\times 10^{6}$
$n_{m}^{\prime }=1.45$, $\mu_{a,m}=150\;m^{-1}$	1.48 × 10^6^	$1.62\times 10^{6}$	$1.68\times 10^{6}$	$1.63\times 10^{6}$	$1.77\times 10^{6}$

**Table 4:** Values of $P_{d}$ (W m^−3^) computed by using $\lambda_{0}=1064$ nm, different models, and different geometrical or constitutive parameters

	Model Figure 4 *t*_m_ = 1 μm	Model Figure 4 *t*_m_ = 3 μm	Model with an isolated mitochondrion	Model Figure 5	Model Figure 7
$n_{m}^{\prime }=1.35$, $\mu_{a,m}=20\;m^{-1}$	$1.83\times 10^{5}$	$1.80\times 10^{5}$	$1.87\times 10^{5}$	$1.85\times 10^{5}$	$1.87\times 10^{5}$
$n_{m}^{\prime }=1.35$, $\mu_{a,m}=85\;m^{-1}$	$7.78\times 10^{5}$	$7.67\times 10^{5}$	$7.93\times 10^{5}$	$7.88\times 10^{5}$	$7.95\times 10^{5}$
$n_{m}^{\prime }=1.35$, $\mu_{a,m}=150\;m^{-1}$	$1.37\times 10^{6}$	$1.35\times 10^{6}$	$1.40\times 10^{6}$	$1.39\times 10^{6}$	$1.40\times 10^{6}$
$n_{m}^{\prime }=1.40$, $\mu_{a,m}=20\;m^{-1}\;$	$1.79\times 10^{5}$	$1.83\times 10^{5}$	$1.91\times 10^{5}$	$1.87\times 10^{5}$	$1.93\times 10^{5}$
$n_{m}^{\prime }=1.40$, $\mu_{a,m}=85\;m^{-1}$	$7.61\times 10^{5}$	$7.80\times 10^{5}$	$8.12\times 10^{5}$	$7.97\times 10^{5}$	$8.22\times 10^{5}$
$n_{m}^{\prime }=1.40$, $\mu_{a,m}=150\;m^{-1}$	$1.34\times 10^{6}$	$1.38\times 10^{6}$	$1.43\times 10^{6}$	$1.41\times 10^{6}$	$1.45\times 10^{6}$
$n_{m}^{\prime }=1.45$, $\mu_{a,m}=20\;m^{-1}$	$1.77\times 10^{5}$	$1.98\times 10^{5}$	$1.96\times 10^{5}$	$1.90\times 10^{5}$	$2.02\times 10^{5}$
$n_{m}^{\prime }=1.45$, $\mu_{a,m}=85\;m^{-1}$	$7.54\times 10^{5}$	$8.40\times 10^{5}$	$8.34\times 10^{5}$	$8.09\times 10^{5}$	$8.59\times 10^{6}$
$n_{m}^{\prime }=1.45$, $\mu_{a,m}=150\;m^{-1}$	$1.33\times 10^{6}$	$1.48\times 10^{6}$	$1.47\times 10^{6}$	$1.43\times 10^{6}$	$1.51\times 10^{6}$

## DISCUSSION

In this work, we made use of suitable models to compute the electromagnetic field inside mitochondria in PBM experiments. Our models took account of the uncertainty on the constitutive parameters, dimensions, and disposition of the mitochondria in the incubation chamber.

For any model, we found all quantities of interest. However, the above uncertainty together with the fact that, on the one hand, every mitochondrion can be different from all others, and that, on the other hand, their states can change with time, reduce the importance of the knowledge of the details of the electromagnetic field inside them and strengthen the significance of average quantities. This is common in electromagnetic dosimetry studies, at all wavelengths [ICNIRP, 2020].

Many of our results related to average quantities were presented in different subsections. Due to their importance, we present here some additional comments on the whole set of results that we computed on the average dissipated power density in mitochondria. An analogous discussion, which is not done due to space considerations, can be performed on the average energy density of the electromagnetic field inside mitochondria.

By considering, in particular, the results obtained when $\mu_{a,m}=85\;m^{-1}$, we observe that:

– At 808 nm, $P_{d}$ belongs to the range [0.822,1.0] MW m^−3^ (having considered different mitochondria dimensions, different values of the real part of their refractive index, different polarizations, different alignments (horizontal or vertical), and different configurations (isolated, layered, clustered)); the mean value is $P_{d,\mathrm{av},808,85}=0.911\;\mathrm{MW}\;m^{-3}$ and the range radius is $R_{d,808,85}=0.089\;\mathrm{MW}\;m^{-3}$, corresponding to 9.8% of the mean value.
– At 980 nm, the range, determined under the same conditions, is [0.624,0.705] MW m^−3^; then $P_{d,\mathrm{av},980,85}=0.664\;\mathrm{MW}\;m^{-3}$ and $R_{d,980,85}=0.040\;\mathrm{MW}\;m^{-3}$ (6.1% of the mean value).
– At 1064 nm, we obtained the range [0.754,0.859] MW m^−3^; therefore $P_{d,\mathrm{av},1064,85}=0.806\;\mathrm{MW}\;m^{-3}$ and $R_{d,1064,85}=0.052\;\mathrm{MW}\;m^{-3}$ (6.5% of the mean value).
– Without any distinction based on the wavelength, $P_{d}$ belongs to [0.624,1.0] MW m^−3^; thus, the mean value and radius become $P_{d,\mathrm{av},85}=0.812\;\mathrm{MW}\;m^{-3}$ and $R_{d,85}=0.188\;\mathrm{MW}\;m^{-3}$ (23.2% of the mean value).

Doing the same for the smallest and largest values of the mitochondria absorption coefficient that we found in the open literature, one can note that:

– At 808 nm, the ranges of $P_{d}$ values are $[0.194,0.225]\;\mathrm{MW}\;m^{-3}$, when $\mu_{a,m}=20$ m^−1^, and $[1.45,1.77]\;\mathrm{MW}\;m^{-3}$, when $\mu_{a,m}=150\;\;m^{-1}$; therefore, $P_{d,\mathrm{av},808,20}=0.209\;\mathrm{MW}\;m^{-3}$, $P_{d,\mathrm{av},808,150}=1.61\;\mathrm{MW}\;m^{-3}$, $R_{d,808,20}=0.015\;\mathrm{MW}\;m^{-3}$ (7.4% of the mean value), and $R_{d,808,150}=\;0.16\;\mathrm{MW}\;m^{-3}$ (9.9% of the mean value).
– At 980 nm, the $P_{d}$ values belong to [0.155, 0.166] MW m^−3^, when $\mu_{a,m}=20\;m^{-1}$, and to [1.16, 1.24] MW m^−3^, when $\mu_{a,m}=150\;m^{-1}$; then $P_{d,\mathrm{av},980,20}=0.160\;\mathrm{MW}\;m^{-3}$, $P_{d,\mathrm{av},980,150}=1.20\;\mathrm{MW}\;m^{-3}$, $R_{d,980,20}\;=0.005\;\mathrm{MW}\;m^{-3}$ (3.4% of the mean value), and $R_{d,980,150}=0.04\;\mathrm{MW}\;m^{-3}$ (3.3% of the mean value).
– At 1064 nm, the ranges are $[0.177,0.202]\;\mathrm{MW}\;m^{-3}$, when $\mu_{a,m}=20\;m^{-1}$, and $[1.33,1.51]\;\mathrm{MW}\;m^{-3}$, when $\mu_{a,m}=150\;m^{‐1}$; thus $P_{d,\mathrm{av},1064,20}=0.189\;\mathrm{MW}\;m^{-3}$, $P_{d,\mathrm{av},1064,150}=1.42\;\mathrm{MW}\;m^{-3}$, $R_{d,1064,20}=0.012\;\mathrm{MW}\;m^{-3}$ (6.6% of the mean value), and $R_{d,1064,150}=0.09\;\mathrm{MW}\;m^{-3}$ (6.3% of the mean value).
– Without any distinction based on the wavelength, $P_{d}$ belongs to $[0.155,0.225]\;\mathrm{MW}\;m^{-3}$, for $\mu_{a,m}=20\;m^{-1}$, and to $[1.16,1.77]\;\mathrm{MW}\;m^{-3}$, for $\mu_{a,m}=150\;m^{-1}$; therefore, $P_{d,\mathrm{av},20}=0.190\;\mathrm{MW}\;m^{-3}$, $P_{d,\mathrm{av},150}=1.46\;\mathrm{MW}\;m^{-3}$, $R_{d,20}=0.035\;\mathrm{MW}\;m^{-3}$ (18.4% of the mean value), and $R_{d,150}=0.30\;\mathrm{MW}\;m^{-3}$ (20.8% of the mean value).

It is interesting to observe that the largest range radius is 23.2% of the mean value of the range for $P_{d}$ values. This significant possible variation of $P_{d}$ was obtained by changing everything (mitochondria dimensions, the value of the real part of their refractive index, polarization, position (horizontal or vertical), configuration (isolated, layered, clustered), and wavelength) apart from the absorption coefficient.

To better appreciate this consideration, one may remember that in practical experiments of PBM, the stability of the power emitted by laser sources can present variations as large as 20% [Pires Oliveira et al., 2008; De Almeida et al., 2012; Brassolatti et al., 2016]. The uncertainty on the absorption coefficient, which can change from 20 to 150 m^−1^, determined much larger variations on the values of $P_{d}$: the overall range was [0.155,1.77] MW m^−3^. The maximum is 11.4 times the minimum, but this is mostly due to the fact that the largest value of the absorption coefficient is 7.5 times the smallest one.

Finally, it could be interesting to observe that for an intermediate value of the absorption coefficient of mitochondria ($\mu_{a,m}=85\;m^{-1}$), the mean of the $P_{d}$ values is $P_{d,\mathrm{av},85}=0.812\;\mathrm{MW}\;m^{-3}$, as indicated above. By referring to the experimental setup to which all our models refer, we have 1 W of incident power (1 W cm^−2^ on an incubation chamber of 1 cm^2^) that illuminates mitochondria occupying a volume of $10^{-10}$ m^3^ ($10^{-4}$ ml). Then we could estimate that overall, they absorb 0.08 mW.

## CONCLUSIONS

Studies on low‐level laser therapy have highlighted the importance of the absorption of the electromagnetic field in the visible or NIR bands by mitochondria. For this reason, in this work, we have considered models, which, referring to a well‐defined experimental layout, allow the evaluation of the electromagnetic field inside the mitochondria. The set of models examined is sufficiently rich to take account of the possible different alignments (horizontal or vertical) and configuration (isolated, layered, or clustered) that mitochondria can assume in practice. Several values of their dimensions and constitutive parameters were considered as well, to take into consideration the differences among mitochondria and the uncertainty of the quantities of interest for electromagnetic models. Finally, different wavelengths and polarizations were used. The effects of the changes of all parameters of our models are presented. Most of the discussion that followed was focused on the average properties of the electromagnetic field inside mitochondria. These results give quantitative estimates of the dosage that the mitochondria are exposed to during PBM experiments and hence can help clear up the inconsistencies that are present in the literature related to those experiments.

The results can be important for understanding the mechanism that causes PBM effects. However, any such consideration on the mechanism of interaction of the electromagnetic field with the multiprotein complexes present inside mitochondria is out of the scope of the present study. Nevertheless, since it provides reliable calculations of the electromagnetic field inside the indicated organelles, we consider it as a first step to deepen our understanding of the mechanism of interaction of interest.
